# Comparison of the Acute Effects of Hold-Relax and Static Stretching among Older Adults

**DOI:** 10.3390/biology10020126

**Published:** 2021-02-05

**Authors:** Masatoshi Nakamura, Shigeru Sato, Ryosuke Kiyono, Kaoru Yahata, Riku Yoshida, Taizan Fukaya, Andreas Konrad

**Affiliations:** 1 Institute for Human Movement and Medical Sciences, Niigata University of Health and Welfare, 1398 Shimami-cho, Kita-ku, Niigata City, Niigata 950-3198, Japan; masatoshi-nakamura@nuhw.ac.jp (M.N.); hpm19006@nuhw.ac.jp (S.S.); hpm19005@nuhw.ac.jp (R.K.); hpm20011@nuhw.ac.jp (K.Y.); fukaya.taizan@gmail.com (T.F.); 2 Department of Physical Therapy, Niigata University of Health and Welfare, 1398 Shimami-cho, Kita-ku, Niigata City, Niigata 950-3198, Japan; hpa17123@nuhw.ac.jp; 3 Department of Rehabilitation, Kyoto Kujo Hospital, 10 Karahashirajoumoncho, Minami-ku, Kyoto 601-8453, Japan; 4 Institute of Human Movement Science, Sport and Health, University of Graz, Mozartgasse 14, A-8010 Graz, Austria

**Keywords:** muscle stiffness, dorsiflexion, ultrasound, ankle plantar flexors

## Abstract

**Simple Summary:**

It is well known that stretching interventions are effective in improving age-related changes in range of motion (ROM) and muscle stiffness. We investigated the effects of various stretching interventions, such as static stretching and hold–relax stretching, on ROM and muscle stiffness in older adults to establish the most effective stretching technique. Our results showed that static stretching and hold–relax stretching increased ROM, which could be contributed by not change in muscle stiffness, but stretch tolerance. Conversely, medial gastrocnemius muscle stiffness decreased only after a static stretching intervention and not after hold–relax stretching. Our results indicated that static stretching intervention improved ROM and muscle stiffness in older adults.

**Abstract:**

Various stretching techniques are generally recommended to counteract age-related declines in range of motion (ROM) and/or increased muscle stiffness. However, to date, an effective stretching technique has not yet been established for older adults. Consequently, we compared the acute effects of hold relax stretching (HRS) and static stretching (SS) on dorsiflexion (DF) ROM and muscle stiffness among older adults. Overall, 15 elderly men and nine elderly women (70.2 ± 3.9 years, 160.8 ± 7.8 cm, 59.6 ± 9.7 kg) were enrolled, and both legs were randomized to either HRS or SS stretching. We measured DF ROM and muscle stiffness using a dynamometer and ultrasonography before and after 120 s of HRS or SS interventions. Our multivariate analysis indicated no significant interaction effects, but a main effect for DF ROM. Post-hoc tests revealed that DF ROM was increased after both HRS and SS interventions. Moreover, multivariate analysis showed a significant interaction effect for muscle stiffness. Post-hoc tests revealed that muscle stiffness was decreased significantly after only SS intervention. Taken together, our results indicated that both HRS and SS interventions are recommended to increase ROM, and SS is recommended to decrease muscle stiffness in older adults.

## 1. Introduction

Generally, range of motion (ROM) is known to decrease with age [1,2], and previous studies have suggested that decreased ROM leads to a decline in locomotion and balance [3,4]. Similarly, a decline in ROM could lead to an increased risk of falls [5]. Accordingly, maintaining and improving ROM among older adults is imperative.

Static stretching (SS) has generally been recommended to counteract age-related decline in ROM. Additionally, previous studies have shown that older adults exhibited an increase in ROM after SS intervention [6,7,8]. SS intervention could also improve mobility tasks, such as the timed up-and-go test and 10-m walk speed test [7,9,10,11]. Moreover, previous studies have shown that increased muscle stiffness associated with antagonist muscle contractions can inhibit joint movement and result in higher energetic/metabolic costs [12,13]. This increase in muscle stiffness among older adults could be a factor inhibiting activities of daily living and might lead to falls in some cases. Although no consensus has been available regarding age-related changes in muscle stiffness [14,15], Nakamura et al. reported that older women experienced a decrease in stiffness (shear elastic moduli) of the medial gastrocnemius (MG) and lateral gastrocnemius (LG) muscle bellies after 300 s of SS intervention [8]. Although Hirata et al. reported no significant changes in the muscle stiffness of the MG and LG muscle bellies among older men after 450 s of SS, they did observe a decrease in muscle stiffness at the distal portion of the MG after the same time of SS [16]. Based on these studies, we can assume that SS intervention increases ROM and decreases muscle stiffness in older adults.

Recently, studies have suggested that the perception of passive tension, called stretch tolerance, could influence ROM. Hirata et al. investigated the association between dorsiflexion (DF) ROM and muscle stiffness or stretch tolerance among both young and older men [17]. Their study showed a significant association between DF ROM and stretch tolerance among both groups of men, suggesting that changing the stretch tolerance considerably increases ROM. Another study showed that a different stretching technique, hold–relax stretching (HRS), had a superior effect in increasing stretch tolerance among young adults compared to SS [18]. Therefore, HRS might be a better technique to enhance ROM in the elderly. To the best of our knowledge, no study has compared the effects of HRS and SS on DF ROM, stretch tolerance, and muscle stiffness among older adults. Therefore, the purpose of this study was to determine and compare the acute effects of HRS and SS on DF ROM, stretch tolerance, and MG muscle stiffness among older adults. Based on the previous study in young adults [18], we hypothesized that HRS shows a superior effect in increasing ROM compared to SS, and that both stretching techniques decrease muscle stiffness among older adults.

## 2. Materials and Methods

### 2.1. Experimental Protocol

**Figure 1:**
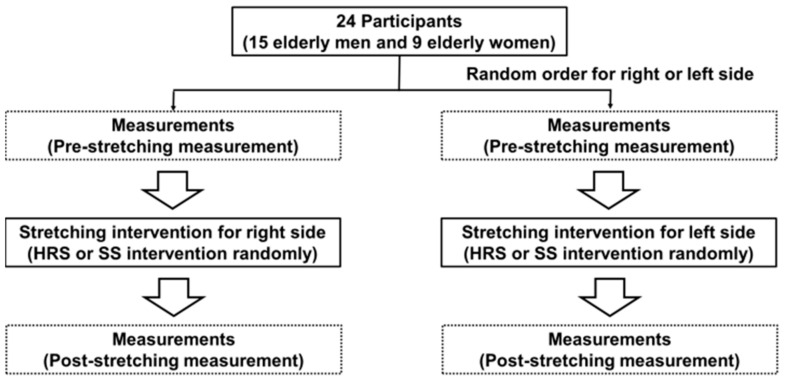
Experimental protocol of this study. HRS: hold–relax stretching, SS: static stretching.

A quasi-randomized experimental design was used to compare the acute effects of HRS and SS on DF ROM and MG muscle stiffness. Both legs were randomly allocated to receive either HRS or SS intervention, and the left and right sides were treated in a random order. All measurements were performed prior to (PRE) and immediately after (POST) HRS and SS. Participants were familiarized with the procedure before measurements and were asked to be relaxed throughout the measurements. In this study, all variables were measured during the passive stretching test (Figure 1).

### 2.2. Subjects

DF ROM and muscle stiffness in both legs were measured in 15 elderly men and nine elderly women. Baseline characteristics (mean ± standard deviation (SD)) were as follows: age, 70.2 ± 3.9 years; height, 160.8 ± 7.8 cm; and body mass, 59.6 ± 9.7 kg. Inclusion criteria were age >60 years, living in the community, and able to walk independently. Exclusion criteria were cognitive impairment, severe cardiac or musculoskeletal disorders, previous diagnosis of pulmonary disease, and hearing impairment. In this study, we used G* power 3.1 software (Heinrich Heine University, Düsseldorf, Germany) for calculating sample size for a split-plot analysis of variance (ANOVA) (effect size = 0.40 [large], α error = 0.05, and power = 0.80). Thus, >22 participants were required for this study. All the participants were informed of the purpose and procedure of the study and gave written informed consent prior to participation. The study was approved by the university (#18193) and performed in accordance with the Declaration of Helsinki.

### 2.3. Assessment of DF Angle and Passive Torque

Participants were instructed to sit on an isokinetic dynamometer (Biodex system 3.0; Biodex Medical Systems, NY, USA) with the hip at 50° flexion and the knee at 0° (i.e., anatomical position). The upper body was fixed using straps, and the ankle was fixed in a footplate at the neutral ankle position. The ankle was then passively moved to the maximum DF position, until the participant experienced discomfort or pain [19,20,21], with an angular velocity of 5°/s. The participants stopped the footplate using motion control. The corresponding angle at this position was defined as the maximum DF ROM. Participants practiced these measurements two to three times before the actual PRE-measurement. The average value of two trials was taken for further analysis. The corresponding passive torque from the ROM assessment was defined as the stretch tolerance [22,23,24]. Participants were asked to be relaxed during this assessment.

Prior to the study, we confirmed that the test–retest reliability for DF ROM, and the passive torque at DF ROM was determined using coefficient variation (CV) and intraclass correlation coefficient (ICC) in 10 young adults (seven male/three female). CV was 6.2 ± 5.0% and 7.1 ± 5.9%, and ICC (1, 2) was 0.903 and 0.937, respectively.

### 2.4. Assessment of Medial Gastrocnemius Stiffness

B-mode ultrasound apparatus (LOGIQ e V2; GE Healthcare Japan, Tokyo, Japan) was used to determine the displacement of the muscle–tendon junction (MTJ) during passive ankle DF. The MTJ was identified and visualized as a continuous sagittal plane on ultrasound image using an 8-MHz linear-array probe. To ensure that the probe did not move during passive DF, a marker was placed on the skin [20,25,26,27], and a custom-made fixation was used. Ultrasound images of the MTJ were quantified using an open-source digital measurement software (Image J, National Institutes of Health, Bethesda, MD, USA). As all participants were able to dorsiflex their ankles more than 15°, the MTJ displacement was analyzed between 0° and 15° of DF. MG muscle stiffness was calculated as the change of the passive torque and MTJ displacement between 0°–15° of DF [20,27]. The average value of two measurements was taken for further analyses.

We confirmed the test–retest reliability for muscle stiffness in 10 young adults (7 male/3 female). CV was 2.8 ± 2.2% and ICC (1, 2) was 0.973.

### 2.5. HRS and SS Techniques

Both HRS and SS were performed using the dynamometer in a sitting position, similar to the previous passive assessments. During HRS, the ankle underwent initially passive DF at a constant velocity of 5°/s, starting from 10° plantar flexion to DF ROM by the dynamometer, after which participants were asked to accomplish submaximal maximal voluntary isometric contraction plantar flexions for 10 s in the same position [28]. After this contraction, the ankle was held at the DF ROM for an additional 20 s. Following each 30-s HRS stretch, the ankle was returned to 10° plantar flexion. The entire 30-s HRS application was then repeated three times, resulting in a total stretching time of 2 min.

During SS, the ankle underwent passive DF, starting from 10° plantar flexion to DF ROM, and was held at the end angle for 30 s. This 30 s SS application was then repeated three times, resulting in a total stretching time of 2 min [18].

### 2.6. Statistical Analysis

SPSS Statistics for Windows version 24.0 (IBM Corp., Armonk, NY, USA) was used for statistical analysis. Differences between HRS and SS techniques at PRE were assessed using the unpaired *t*-test. A two-way ANOVA with time (PRE vs. POST) and technique (HRS vs. SS) was used for multivariate comparison. Furthermore, a post-hoc paired t-test was used to determine differences between PRE and POST in each technique. Differences were considered significant at an alpha level of 0.05. Effect sizes (ES) were calculated and interpreted according to the suggestions of Cohen [29]. If ANOVA showed a significant interaction or main effect, a pairwise comparison was conducted using paired t-test with Bonferroni correction. The linear relationships of the various parameters were tested with. Data is presented as means ± SD.

## 3. Results

PRE and POST values during HRS and SS are summarized in Table 1. No significant differences in PRE values were observed between both techniques between the right and left legs. ANOVA indicated no significant interaction effects for DF ROM and passive torque at DF ROM (F = 0.85, *p* = 0.362, ηp2 = 0.02 and F = 0.35, *p* = 0.555, ηp2 = 0.01, respectively), but a significant main effect was seen for DF ROM (F = 64.6, *p* < 0.01, ηp2 = 0.584). Post-hoc test revealed that POST DF ROM values were significantly higher than PRE values in both techniques (HRS: *p* < 0.01, ES = 0.51 and SS: *p* < 0.01, ES = 0.42, respectively). Moreover, split-plot ANOVA indicated a significant interaction effect for muscle stiffness (F = 4.55, *p* = 0.038, ηp2 = 0.09). Post-hoc test revealed that POST muscle stiffness values were significantly lower than PRE values during SS (*p* < 0.01, ES = 0.87), whereas no significant difference was observed during HRS (*p* = 0.220, ES = 0.23).

**Figure 2:**
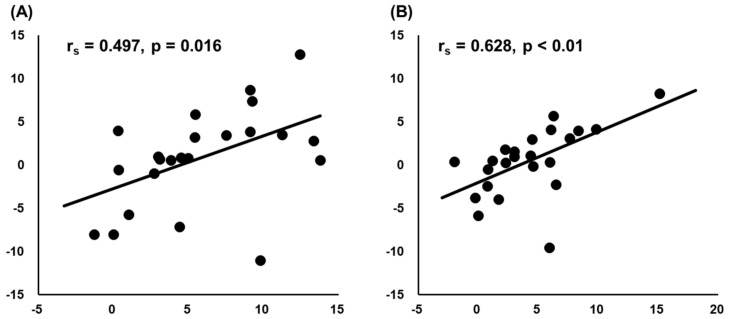
Relationship between change in dorsiflexion range of motion (DF ROM) and change in passive torque at DF ROM after hold–relax stretching (**A**) and static stretching (**B**).

In addition, Spearman’s rank correlation coefficients showed a significant correlation between changes in DF ROM and changes in passive torque at DF ROM after HRS and SS (Figure 2: r_s_ = 0.497, *p* = 0.016 and r_s_ = 0.628, *p* < 0.01, respectively). However, no significant correlation was observed between changes in DF ROM and changes in muscle stiffness (HRS: r_s_ = −0.17, *p* = 0.44 and SS: r_s_ = −0.214, *p* = 0.72).

## 4. Discussion

We investigated the acute effects of HRS and SS on DF ROM, stretch tolerance, and MG muscle stiffness among community-dwelling older adults. Our results showed that DF ROM increased significantly after both HRS and SS with no significant differences being observed between both interventions. In addition, muscle stiffness was decreased after only SS. Although previous studies had investigated the acute effects of HRS and SS on DF ROM and muscle stiffness in young adults [18,30], the current study is, to the best of our knowledge, the first study to investigate the acute effect of HRS and SS on DF ROM and muscle stiffness among older adults.

Our study showed that DF ROM increased significantly after both HRS and SS, which is consistent with previous studies on young [18,31,32] and older adults [33,34]. Moreover, split-plot ANOVA indicated no significant interaction effects for DF ROM, which suggested that increases in DF ROM did not significantly differ between HRS and SS. Interestingly, studies have suggested that the increase in ROM following HRS and SS may differ depending on the target muscle. Previous studies, which compared the effects of different stretching intervention methods on the hamstring muscle, showed that HRS promoted a greater increase in ROM rather than SS [35,36,37]. Conversely, previous studies investigating the effects of stretching intervention on the gastrocnemius muscle, similar to the current study, showed no significant difference between HRS and SS in terms of increase in ROM [18,31,32]. In addition, previous studies comparing the acute effects of HRS and SS among older adults have shown no significant differences in the increase in ROM between HRS and SS [33,34]. Taken together, the increment effects of HRS and SS intervention on ROM might perhaps differ according to target muscle or age group. Consequently, more evidence is needed to clarify the differences in the effects of HRS and SS based on target muscle and population.

ANOVA indicated a significant interaction effect for muscle stiffness. Moreover, the post-hoc test revealed that muscle stiffness decreased following SS (*p* < 0.01, ES = 0.87), whereas no significant difference had been noted during HRS (*p* = 0.220, ES = 0.23). This can be underlined with the findings of further studies, who reported that muscle stiffness among older adults decreased after SS [8,16]. Sobolewski et al. (2014), who investigated the acute effects of a 120-s passive stretching on viscoelastic responses among young and older men, showed that changes in viscoelastic properties were similar between young and older men [38]. Moreover, after investigating the effects of a 300-s SS on muscle stiffness among young and older women, Nakamura et al. revealed no differences in the acute effects of SS on muscle stiffness between young and older women [8]. As discussed previously, little difference in the acute effects of SS on muscle stiffness may perhaps exist between young and older adults. On the other hand, Nakamura et al. reported that muscle stiffness decreased after HRS among young adults [18], which was inconsistent with the results of current study. We believe that this discrepancy may involve differences in muscle elongation duration during HRS. The duration of muscle elongation in this study was 80 s (20 s × 4 repetitions), whereas the duration of muscle elongation in the previous study was 100 s (25 s × 4 repetitions). Although further studies are needed to investigate the effects of different muscle elongating durations during HRS intervention on muscle stiffness, we assumed that the HRS protocol used herein did not have sufficient muscle elongation duration to decrease MG stiffness among older adults.

Regarding the increase in DF ROM, Spearman’s rank correlation coefficient showed significant associations between the changes in DF ROM and the changes in passive torque at DF ROM after both HRS and SS. Conversely, no significant associations had been observed between the changes in DF ROM and change in muscle stiffness. Our results support those of Kay et al., who investigated the effects of HRS and SS among young adults [39]. On the other hand, Nakamura et al. (2015) reported that HRS promoted a greater change in stretch tolerance than SS, whereas SS promoted a greater decrease in muscle stiffness than HRS in young adults [18]. Moreover, one previous study suggested that the effects of stretching on end ROM may be related to the decrease in muscle stiffness and modifications in stretch tolerance [22]. However, although MG stiffness decreased after SS intervention in the present study, the only significant association observed was that between the changes in DF ROM and the changes in stretch tolerance. Our results supported those of Hirata et al. (2020), which showed a significant association between DF ROM and stretch tolerance among older men [17]. Although the mechanisms for the change in stretch tolerance after stretching intervention have currently been unclear, our results showed that HRS and SS were effective in changing DF ROM among older adults, while SS was effective in decreasing MG stiffness among older adults.

As outlined, age-related ROM decline leads to a decline in locomotion and balance [3,4] and an increased risk of falls [5]. Therefore, both HRS and SS intervention could increase ROM in older adults, improve locomotion and balance functions, and decrease the risk of falls. In addition, previous studies have shown that increased muscle stiffness associated with antagonist muscle contractions inhibits joint movement, and may result in higher energetic/metabolic costs [12,13]. Our results indicated that SS intervention decreased muscle stiffness in older adults; therefore, it could improve joint movement and energetic/metabolic costs. Our results recommend both HRS and SS interventions to increase ROM and SS to decrease muscle stiffness in older adults.

Our study had some limitations. First, we investigated only acute effects of HRS and SS on gastrocnemius muscles in older adults. Second, the effects of stretching interventions on locomotion, balance function, and risk of falls were not investigated in this study. Therefore, future studies should investigate the chronic effect of HRS and SS intervention on ROM and muscle stiffness as well as on physical functions and the risk of falls in older adults.

## 5. Conclusions

The current study investigated the acute effects of 120-s HRS and SS interventions on DF ROM, stretch tolerance, and muscle stiffness of the MG among older adults. Accordingly, our results showed that both HRS and SS intervention were effective in improving DF ROM, with the change in DF ROM being related to the change in stretch tolerance. On the other hand, our results suggested that SS was required to decrease MG stiffness instead of HRS.

## Figures and Tables

**Table 1:** Changes in variables before and after stretching interventions.

	SS		HRS	
	PRE	POST	PRE	POST
DF ROM (°)	30.9 ± 10.0	35.3 ± 10.8 **	31.7 ± 10.6	37.2 ± 11.1 **^,#^
Passive torque at DF ROM (Nm)	27.1 ± 14.1	27.7 ± 12.4	28.3 ± 12.0	29.7 ± 10.5
Muscle stiffness (Nm/cm)	17.3 ± 14.1	8.4 ± 6.3 **	17.3 ± 11.4	14.6 ± 11.8 ^§^

^#^ = significant main effect. ^§^ = significant interaction effect. ** *p* < 0.01; significantly different from PRE. SS, static stretching; HRS, hold–relax stretching; PRE, before stretching; POST, after stretching; DF, dorsiflexion; ROM, range of motion.

## Data Availability

All data generated or analyzed during this study are included in this published article.
